# Exploring preference for delivery methods for a psychosocial intervention for prenatal anxiety: A qualitative study from a tertiary care hospital in Pakistan

**DOI:** 10.1017/gmh.2024.59

**Published:** 2024-05-09

**Authors:** Maria Atiq, Huma Nazir, Atif Rahman, Abid Malik, Najia Atif, Pamela J. Surkan

**Affiliations:** 1 Human Development Research Foundation, Gujar Khan, Pakistan; 2 Institute of Population Health, University of Liverpool, Liverpool, UK; 3 Public Mental Health Department, Health Services Academy, Islamabad, Pakistan; 4 Department of International Health, Johns Hopkins University Bloomberg School of Public Health, Baltimore, MD, USA

**Keywords:** prenatal anxiety, delivery methods, COVID-19 pandemic, family support, logistical challenges

## Abstract

**Objective:**

This qualitative study explores therapists’ and participants’ preferences for delivery methods (face-to-face and phone sessions) of a cognitive behavioral therapy-based psychosocial intervention for prenatal anxiety delivered in a tertiary care hospital.

**Setting:**

The research was conducted in a randomized controlled trial in Pakistan, where a shift from face-to-face to phone-based therapy occurred during the coronavirus disease-2019 (COVID-19) pandemic.

**Participants:**

Twenty in-depth interviews and a focus group discussion were conducted with participants and therapists, respectively. Transcripts were analyzed using thematic analysis.

**Results:**

Participants generally preferred face-to-face sessions for rapport building, communication, and comprehension. However, barriers like venue accessibility, childcare, and lack of family support hindered engagement. Telephone sessions were favored for easy scheduling and the comfort of receiving the session at home, but there were challenges associated with phone use, distractions at home, and family members’ limited mental health awareness. A mix of face-to-face and telephone sessions was preferred, with rapport from in-person sessions carrying over to telephone interactions.

**Conclusion:**

This study underscores the need for adaptable intervention delivery strategies that consider cultural norms, logistical challenges, and individual family dynamics. By combining the benefits of both delivery methods, mental health interventions can be optimized to effectively address prenatal anxiety and promote well-being in resource-constrained settings like Pakistan.

## Impact statement

This study sheds light on the crucial considerations surrounding the delivery methods of psychosocial interventions, that is, face-to-face vs. phone-based, in settings where mental health facilities are scarce or not easily accessible. The findings emphasize the importance of adaptable intervention delivery strategies that take into account cultural norms, logistical challenges, and family dynamics.

## Introduction

Traditionally, psychotherapies and counseling have been conducted through face-to-face interactions (Zeren, 2015). However, the global coronavirus disease-2019 (COVID-19) pandemic necessitated a rapid transition to online therapy, with little time for many therapists to prepare (Békés and Aafjes-van Doorn, 2020). Remote delivery of psychotherapy has been advocated as an alternative to enhance accessibility to psychological treatments (Barceló-Soler et al., 2023). The Swedish Council on Health Technology Assessment highlights that online therapy is cost- and time-efficient (Bengtsson, 2015), offering a convenient means to reach individuals who may either avoid or face difficulties in receiving treatment at a clinic (Gratzer and Khalid-Khan, 2016). Nevertheless, online therapy poses several challenges in its implementation, including difficulties in interpreting facial expressions or body language, effectively responding to crises, maintaining confidentiality (Kotera et al., 2021), and in reaching individuals with limited access to telephones or computers (Bengtsson, 2015).

An international longitudinal survey of 1,257 therapists conducted during the transition from in-person to online therapy at the onset of the COVID-19 pandemic, as well as 3 months later, identified four types of challenges (Békés and Aafjes-van Doorn, 2020). First, there were challenges related to emotional connection, wherein therapists faced difficulties in establishing a sense of connection with patients, accurately understanding their emotions, and expressing or feeling empathy. Second, distractions were found to be a problem, with therapists and patients becoming sidetracked during therapy sessions. Third, privacy concerns arose, including issues such as finding a suitable private space for therapy. Lastly, therapists experienced difficulties in maintaining professional boundaries and establishing a conducive online workspace. Qualitative studies have yielded mixed findings (Kotera et al., 2021), with some clients perceiving online therapy as impersonal, lacking authenticity, and creating barriers to establishing a therapeutic alliance. These experiences may also be influenced by cultural norms and the recipients’ socioeconomic status, indicating that the online delivery of psychotherapy is a multifaceted and intricate process.

Perinatal anxiety poses a notable challenge in low- and middle-income countries (LMICs), affecting one out of every four women either during pregnancy or in the postpartum period. The pooled estimates of anxiety symptoms in LMICs are 29.2% during the antenatal period and 24.4% during the postnatal period (Nielsen-Scott et al., 2022). The Happy Mother-Healthy Baby (HMHB) study specifically addresses the issue of prenatal anxiety in LMICs, emphasizing the necessity for targeted treatment (Surkan et al., 2020). Levels of prenatal anxiety and postnatal depression are very high in Pakistan (Waqas et al., 2015), making it an ideal setting for such a study. Also, specialized mental health professionals are scarce, suggesting the need for effective programs that can be implemented by non-specialist providers (Saxena et al., 2007). Therefore, recognizing the importance of mental health care during pregnancy, especially in this region, we conducted a phase 3, two-arm, single-blind, randomized controlled trial (RCT) in Pakistan with non-specialized providers. This anxiety-focused early prenatal preventive intervention was effective in reducing the prevalence of postnatal common mental disorders (CMDs; Surkan et al., 2024). This research contributes to the evidence base for advancing interventions that can alleviate the burden of mental health challenges in the perinatal period in resource-constrained settings.

In Pakistan, the closure of hospital outpatient departments due to the COVID-19 pandemic prompted a shift from face-to-face to phone-based intervention delivery in the HMHB study. To date, no research has been conducted to compare the experiences of online and face-to-face therapy in Pakistan, where a massive mental health treatment gap exists due to a scarcity of mental health professionals and facilities (Jacob et al., 2007). Conducting such studies can provide valuable insights into leveraging existing resources effectively to address this gap.

This transition provided us with a unique opportunity to conduct an in-depth investigation into the experiences of both participants and research therapists who encountered both face-to-face and phone therapy, among a primarily low-income population of pregnant women in Pakistan. Consequently, this study aimed to explore the experiences associated with the delivery and receipt of a cognitive behavioral therapy (CBT)-based treatment for prenatal anxiety using both phone-based and face-to-face methods.

## Methods

### Research design

A qualitative research design was used in this study as it allowed the researchers to explore in-depth experiences, behaviors, and perspectives of participants and to generate insights that can inform theory, practice, policy, and increase the credibility of the data.

### Settings and participants

This study was nested within an RCT that was conducted at a tertiary care hospital in Rawalpindi, Pakistan. It aimed to evaluate a psychosocial intervention called HMHB for prenatal anxiety. The trial took place from April 16, 2019, to October 7, 2022, and was registered with the US National Library of Medicine (clinicaltrials.gov identifier NCT03880032; Surkan et al., 2020). Participants were recruited from the Obstetrics and Gynecology Department of a large public hospital. Participant inclusion criteria included: ≤ 22 weeks of gestation, aged ≥18 years, residing within a 20 km radius of the study hospital with the intention to remain in the area until the study’s completion, ability to understand spoken Urdu and a score of ≥8 on the Hospital Anxiety and Depression Scale (HADS; Zigmond and Snaith, 1983). Exclusion criteria included a diagnosis of depression according to the Structured Clinical Interview for DSM Disorders (SCIDs; First and Gibbon, 2004; Gorman et al., 2004).

Participants in this qualitative study were purposively recruited between March 2021 and August 2021, from the arm that received the intervention. We sampled participants to achieve variation on the number of sessions they received in-person and over the telephone, as well as their age, education, and number of children. All participants in this study received at least the first session in-person. After sampling, the participants were contacted over the phone to inquire if they were interested in participating in an interview. Informed consent was obtained upon receiving a positive response, prior to conducting the interview. Initially, all HMHB participants recruited, received face-to-face sessions. However, in response to the COVID-19 pandemic, lockdown measures were implemented on March 24, 2020, and lasted until August 17, 2020, leading to the closure of hospital outpatient departments (Anadolu Agency, 2020). During the lockdown period, all the sessions were conducted over the telephone. After the lockdown, as the screening resumed, existing and new participants were given the option to receive sessions either face-to-face or by telephone. This decision was made to address participants’ ongoing apprehension about coming to the hospital due to COVID-19, even after the official lockdowns were over.

Detailed participant demographic information and the number of sessions delivered through each modality can be found in Table 1.

**Table 1.** Characteristics of the participants

Reference number	Age	Years of schooling	Number of children	Family structure	Core sessions received in-person (out of six)	Core sessions received over the phone (out of six)
MO1	34	12	4	Nuclear	1	5
MO2	26	12	2	Nuclear	4	2
M03	24	14	2	Joint	4	2
M04	20	10	1	Joint	1	2
M05	25	12	3	Nuclear	5	1
M06	26	10	3	Nuclear	4	2
M07	43	14	7	Joint	2	4
M08	33	8	1	Joint	5	1
M09	42	8	3	Nuclear	3	3
M10	37	5	1	Joint	3	3
M11	27	4	2	Joint	3	1
M12	23	12	2	Joint	3	3
M13	29	10	3	Nuclear	4	1
M14	28	0	3	Joint	5	1
M15	30	8	4	Nuclear	4	2
M16	26	10	2	Joint	5	1
M17	27	0	3	Nuclear	5	1
M18	34	10	5	Nuclear	3	3
M19	30	5	3	Nuclear	3	3
M20	24	10	1	Joint	5	1

### Data collection

Data were collected from trial participants in the intervention arm and the research therapists who delivered the intervention. Research therapists were non-specialist providers with a 2-year bachelor’s degree and 2-year master’s degree in psychology and no clinical experience. They were trained in delivery of HMHB by the mental health specialist (NA). The data collection included in-depth interviews (IDIs) with 20 women and a focus group discussion (FGD) with seven therapists. Out of the 20 IDIs, 11 were conducted face-to-face, while nine were carried out over the phone. A topic guide was developed, pilot-tested with two women, and revised based on the feedback received. The interviews were scheduled at times that were convenient for the participants, and measures were taken to ensure privacy and confidentiality. The IDIs were conducted by two research associates who had prior experience in collecting qualitative data. Face-to-face interviews took place in a private room in the department of Obstetrics and Gynecology at Holy Family Hospital and lasted between 40 and 60 min. During face-to-face interviews specifically, strict protocols were implemented to ensure that only the participant, note-taker, or interviewer was present in the room during data collection. This setup was intentionally designed to create a secure environment, fostering a sense of safety for participants to respond and speak freely without hesitation. Prior to conducting telephone interviews, the interviewers received specific training for conducting interviews over the phone, focusing on the importance of addressing participants’ privacy concerns and implementing mitigation strategies when necessary. These strategies included advising interviewers to guide the conversation away from sensitive topics or to disconnect the call if they felt a participants’ privacy was compromised. Additionally, prior to initiating each interview, interviewers reiterated the confidentiality policy. Notably, interviewers were trained to promptly cease the interview if a participant was at risk of self-harm or posing harm to others, followed by conducting a risk assessment.

To safeguard the data, audio-recorded files were stored on secured computers, and hard data related to participants or interview transcripts were kept in a locked cabinet. Only authorized personnel from the research team had access to the data.

### Content of the HMHB intervention

HMHB is a CBT-based psychosocial intervention aimed at treating prenatal anxiety (Atif et al., 2020). It was adapted from the Thinking Healthy Program (THP), an evidence-based psychosocial intervention for mothers experiencing perinatal depression (Rahman et al., 2008). It consists of six core sessions and up to six additional booster sessions, which are conducted individually by a trained research therapist under the supervision of a mental health specialist. The first five core sessions are delivered weekly, starting as early in the pregnancy as possible. These are followed by the booster sessions, the frequency and duration of which are tailored to the individual needs of the woman and integrated into her routine visits. The final core session is delivered late in the third trimester of pregnancy. The core components of the intervention involve establishing an empathetic relationship, challenging negative thoughts, promoting active behavior, problem-solving, and encouraging family involvement (Atif et al., 2020). HMHB focuses on enhancing the well-being of mothers in three key areas: their personal health, their relationships with significant others, and their bond with their baby. To effectively engage participants, the program uses culturally tailored illustrations and scenarios that raise mental health awareness, facilitate cognitive restructuring, and collaboratively set tasks for behavioral activation. It also involved relaxation exercises for managing anxiety symptoms (Atif et al., 2020). Finally, after the final core session, up to six booster sessions were offered to participants who were deemed to need reinforcement.

### Data analysis

Data collection and analysis were carried out simultaneously. Thematic analysis, a method that emphasizes identifying, analyzing, and interpreting patterns of meaning within qualitative data (Clarke and Braun, 2013), was employed for data analysis. All six steps of thematic analysis were used: (1) becoming familiar with the data, (2) generating codes, (3) searching for themes, (4) reviewing themes, (5) defining and naming themes, and (6) writing up (Maguire and Delahunt, 2017). After transcribing each interview, the raw data were read and re-read to gain familiarity and to generate codes. A code sheet was developed and thoroughly examined to establish connections and associations. This process facilitated the emergence of subthemes and themes, as presented in Table 2.

**Table 2.** Themes and subthemes related to delivery of Happy Mother–Healthy Baby in-person versus by phone

Themes	Subthemes
Theme 1: Preference for face–to–face sessions over phone sessions	*1.1: Better rapport building*
	*1.2: Better nonverbal and verbal communication*
	*1.3: Better comprehension of intervention contents and activities*
	*1.4: Conducive therapeutic environment*
	*1.5: Better family involvement*
Theme 2: Barriers to face–to–face sessions in comparison to phone sessions	*2.1: Travel to the venue*
	*2.2. Preoccupation with children and competing demands from family*
Theme 3: Preference for telephone sessions in comparison to face–to–face sessions	*3.1: Better flexibility of scheduling sessions*
	*3.2: Less hassle and fewer costs*
	*3.3: Less hesitance to talk over the phone*
Theme 4: Barriers to phone sessions in comparison to face–to–face sessions:	*4.1: Unavailability of phone and poor connectivity*
	*4.2: Loss of participant interest/concentration*
	*4.3: Lack of mental health awareness*
Theme 5: Preference for mixed therapy	*5.1: Initial face–to–face sessions allow rapport building*
	*5.2: Handing over of the intervention material*

Reflexivity was maintained through consistent field notes and bi-weekly discussions among team members and with the supervisor. To mitigate bias, interview transcripts were also shared and discussed among the research team and with the supervisor. In the analysis, we made both horizontal and vertical comparisons, that is, comparing findings across different participants’ transcripts as well as within an individual’s transcript. Furthermore, all steps of thematic analysis were carefully followed including reading and re-reading the transcripts and analyzing and discussing any outliers with the team, ensuring transparency and accuracy in our findings.

Ethical approval for this research was obtained from the Institutional Review Boards of Rawalpindi Medical University, the Human Development Research Foundation, the Johns Hopkins Bloomberg School of Public Health, and a National Institute of Mental Health (NIMH) appointed Data Monitoring and Safety Board. All participants provided written informed consent before data collection.

## Results

In total, 20 IDIs (11 in-person and 9 by phone) were conducted with participants from the intervention arm. We also carried out an FGD with seven therapists. Interviews were conducted in-person or over the telephone based on participants’ preferences. All participants had received at least one telephone session of the HMHB intervention. Participants were between 24 and 43 years old, with a mean age of 29.4. They had between 0 and 14 years of formal schooling, and between one and five children (see Table 1). The analysis of the data generated five themes with two to five subthemes grouped under each theme (see Table 2).

### Theme 1.0: Preference for face-to-face sessions over phone sessions

#### Subtheme 1.1: Better rapport building

Most of the participants expressed appreciation for having face-to-face sessions, highlighting positive impact of these sessions on their relationship with their therapists. They valued the empathetic behavior of their therapists and felt a sense of hope. One participant described her experience, saying, “It was my first session; I remember looking at my therapist. Her voice was so reassuring. She told me that if I tried, I would get better bit by bit. She demonstrated the breathing exercises in front of me, and then we practiced together. I left the session feeling so calm” (IDI-M12).

Confidentiality was another factor that participants valued when expressing a preference for face-to-face sessions. They developed a greater sense of trust in their therapists and experienced increased reassurance regarding the confidentiality of their discussions. One participant explained, “I shared all my worries, things I hadn’t told anyone before. I knew it would not be disclosed. Over the phone, it was different; God knows there might be another person sitting in the room with her” (IDI-M01).

#### Subtheme 1.2: Better nonverbal and verbal communication

The therapists believed that face-to-face sessions allowed them to pick up on participants’ nonverbal cues, which assisted in their therapeutic work. One therapist highlighted the advantages of being in-person with participants, stating, “During a face-to-face (session), you can tell how participants are feeling just by looking at their facial expressions, which helps in therapy. You can also tell while reviewing their charts whether they have actually practiced their homework or not, something which was hard to guess over the phone” (FGD-R1).

Another participant, who had mostly attended face-to-face sessions, stated, “During the face-to-face sessions, I felt relaxed and shared all my worries with my therapist. My last session was on the phone; it was different talking without seeing her. I could have shared much more if it had not been a phone session” (IDI-M16).

#### Subtheme 1.3: Better comprehension of intervention contents and activities

For the therapists, face-to-face sessions provided a valuable opportunity to comprehend the intervention contents and strategies effectively. They could use illustrations to visually convey information and engage in discussions with the participants. Moreover, in-person they were able to demonstrate breathing and relaxation exercises and set goals for the participants using health charts. They found that explanations of intervention activities over the phone were not fully comprehended by some participants; “There were some exercises that were difficult to understand. Doing these exercises in-person would have been easier” (IDI-M01).

The therapists also found it easier to discuss sensitive topics like domestic abuse and gender preference when they were physically present with the participants. One therapist shared her perspective, saying, “Face-to-face sessions made it easier to explain the intervention’s contents and review the health charts. Also, talking about sensitive topics like domestic violence was less intimidating” (FGD-R3).

#### Subtheme 1.4: Conducive therapeutic environment

Many participants, who belonged to lower socioeconomic status families and lived in overcrowded houses, preferred the hospital setting as it offered them some breathing space and a sense of privacy. They perceived it as an opportunity to take a break from their daily responsibilities. One participant expressed, “I would prefer face-to-face sessions. At home, we have a lot of household responsibilities, but when we come here (to the hospital), we felt that we had some time for ourselves too” (IDI-M04 (DO)). Furthermore, most participants felt at ease during face-to-face sessions, as it allowed them to talk openly without the fear of being overheard. A participant shared her experience, saying, “During face-to-face sessions, we used to talk openly without any hesitation or fear. For phone sessions, I used to sit in a separate room while the family was in the other room. However, I could hear them roaming around outside the room, and that bothered me” (IDI-M12). Some participants also expressed discomfort during phone sessions fearing that their children, going back and forth into the room, might overhear the conversation.

#### Subtheme 1.5: Better family involvement

The intervention encouraged key family members to attend the sessions to enhance their support for the participants, which was reported by the therapists as beneficial for most participants; “In most cases when husbands and mothers-in-law attend the session with the participants, they realize its importance and encourage them to attend sessions regularly” (FGD-R2). A therapist further added that involving the family members was much easier during the delivery of face-to-face sessions; “Over the phone sometimes they say, ‘yes my husband is also listening to you’, but without directly interacting with him, you can’t tell how attentive or engaged he was during the session” (FGD-R2).

### Theme 2.0: Barriers to face-to-face sessions in comparison to phone sessions

#### Subtheme 2.1: Travel to venue

Two significant barriers to accessing the venue were identified by the participants. First, some participants faced challenges due to their families’ custom to be accompanied by either a male family member or a female elder. Consequently, the absence of an escort resulted in their missed appointments. For instance, a 26-year-old participant shared her disappointment: “I struggled to attend face-to-face sessions; my husband was at work, and no one else was available to accompany me to the hospital” (IDI-M06).

Second, participants who lived farther away from the hospital encountered difficulties in affording the travel expenses for attending the weekly sessions. Despite receiving a standard travel allowance of $2 US, one participant described the situation, stating, “My husband is a very angry person. He used to fight with me about the travel expenses to attend appointments. Attending sessions at the hospital became difficult for me because of our financial problems” (IDI-M15).

#### Subtheme 2.2: Preoccupation with children and competing demands from family

Participants who had young children expressed difficulty in fully concentrating during the sessions due to their worries about their children left at home. A mother of three explained, “It was difficult for me to leave my children at home. It took me an hour to reach the hospital and another hour to receive the session. I used to worry about them while receiving the session” (IDI-M17). Similarly, a few participants expressed that during in-person sessions they experienced competing demands on them from their families. A participant who dropped out after receiving three sessions stated, “It was difficult to attend sessions in the hospital because my in-laws started calling me on the phone and asking me to come home, and my husband also used to say that it is getting late, and I should be back” (IDI-M04 (DO).

### Theme 3.0: Preference for telephone sessions in comparison to face-to-face sessions

#### Subtheme 3.1: Better flexibility in scheduling sessions

The participants who were experiencing issues in accessing the face-to-face sessions found the phone sessions more manageable and convenient. They appreciated the flexibility it offered in terms of scheduling. One participant explained, “My therapist would adjust session times for my convenience. This approach allowed me to successfully receive my sessions” (IDI-M01). Similar views were expressed by a therapist, “Some participants found telephone sessions helpful as they didn’t need to travel to the hospital. Moreover, they felt more comfortable receiving sessions at home” (FGD-R1).

#### Subtheme 3.2: Less hassle and fewer costs

One of the major reasons most participants preferred telephone sessions was the convenience of receiving them without disrupting their daily routine. A participant stated, “Sessions over the telephone were good. I used to take sessions from home, whereas for the face-to-face sessions, I would have to take time out from household responsibilities to go to the hospital” (IDI-M17). Another reason for preferring phone sessions was the time and cost savings associated with not having to travel. As one participant mentioned, “The time that would have been wasted in traveling was saved. During that time, I used to do my housework” (IDI-M03). A participant reported that her husband also expressed a preference for her to have the HMHB sessions over the phone: “My husband used to say that phone sessions are better because the travel expenses are hard to bear, and it’s also saved him from the trouble of looking after the children” (IDI-M15).

#### Subtheme 3.3: Less hesitance to talk over the phone

The intervention aims to encourage participants to openly share their emotional problems. For a few participants who were reluctant to talk in-person, telephone sessions provided a more useful and easier means of communication. One participant shared her views saying, “Some people can’t talk openly face-to-face, so they can talk more easily on the phone” (IDI-M02). This sentiment was supported by a therapist who reported, “One of my participants said that she feels more comfortable at home and talking over the phone, as she felt hesitant to share her problems sitting in-person with her therapist” (FGD-R2).

### Theme 4.0: Barriers to phone sessions in comparison to face-to-face sessions

#### Subtheme 4.1: Unavailability of phone and poor connectivity

Many women did not have personal phones and had to share or borrow from their husbands or other family members. This led to delays in receiving sessions, as mentioned by a participant, “I used to take calls on my husband’s mobile. Sometimes he was at home and sometimes not, so my sessions got delayed” (IDI-M02). Therapists also faced challenges in reaching participants, often having to make multiple calls or wait for extended periods of time to connect with the participants, as explained by a therapist, “It wasn’t easy for us to deliver sessions to participants who didn’t own a cellphone. We used to call their family members’ phones and at times were put on hold for a long period of time” (FGD-R3).

Some participants experienced difficulties due to electricity failures or poor internet signals, resulting in frequent dropped calls during the sessions. One participant, who eventually stopped receiving the intervention, shared her experience, saying, “I faced difficulty in receiving sessions. Because of the poor connection, the call used to drop so many times. She [the therapist] used to call me back again and again to complete the session” (IDI-M04 (DO). Therapists also expressed frustration due to the impact of poor telephone connections, which could lead to participants losing interest or engagement in the program.

#### Subtheme 4.2: Loss of participants’ interest/concentration

The therapists reported the duration of phone sessions was much shorter compared to face-to-face and complained that participants sometimes either lost interest or struggled to concentrate, resulting in brief responses. One therapist expressed, “It was difficult for me to explain things over the phone. Some participants struggled to understand me; some used to make excuses by saying, ‘I had lost my health file or I couldn’t find the right page’” (FGD-R3). Similar views were expressed by a participant stating, “I used to talk briefly on the phone. I lost track of our conversation and couldn’t talk in as much in detail” (IDI-M02).

Furthermore, the therapists felt that participants valued face-to-face sessions more compared to telephone sessions, “We felt that they were not valuing our phone services. At times they didn’t respond to our calls or told us to call back later because they were busy” (FGD-R4).

#### Subtheme 4.3: Lack of mental health awareness

Most participants described a lack of awareness and empathy from their in-laws regarding talking therapy. A therapist recalled a participant expressing, “My mother-in-law can’t understand why the therapist is calling me at home and why am I talking about my personal issues with a stranger” (IDI-M16). Another participant complained about being scolded by her mother-in-law: “She used to taunt me by saying that she’d been pregnant a few times, but never had undergone phone consultations with doctors, like I was” (IDI-M06). A few participants mentioned family members coming into the room out of curiosity to find out what they were talking about over the phone.

### Theme 5.0: Preference for mixed therapy

#### Subtheme 5.1: Initial face-to-face sessions allow rapport building

Overall, mixed delivery of therapy was favored by most participants, as it combined the strengths of both delivery methods. A participant living in a nuclear family stated, “In my experience, the mixed approach is quite valuable as it gave me options (to receive sessions either face-to-face or over the phone). It also appealed to me because my therapist listened attentively during both face-to-face and phone interactions” (IDI-M18). A majority of those who showed their preference for mixed therapy expressed the importance of having at least the first session conducted face-to-face. They felt that the initial in-person interaction allowed them to build rapport with their therapists, understand the basics of the intervention, and grasp what was expected from them, along with receiving the intervention materials. A participant expressed, “My first and second sessions were face-to-face. If not for the face-to-face sessions, I don’t think I would have that relationship with my therapist or have understood what was being explained” (IDI-M06).

Therapists also emphasized the importance of building a relationship in-person first before switching to phone sessions. A therapist reported, “Having met them once or twice before the phone sessions really helped me to build our relationship, understand their problems, explain the health charts, and demonstrate the breathing exercises” (FGD-R1).

#### Subtheme 5.2: Handing over of intervention material

The initial face-to-face session/s allowed the therapists to hand the intervention materials (such as health charts to indicate their activities and progress with the intervention) to the participants and to demonstrate the relaxation exercises. These interactions were reported to be very helpful for the majority of the participants. They found that doing the exercises initially in-person with the therapists allowed them to fully comprehend them and to continue practicing them afterward. Furthermore, the intervention materials that were given to them could be referred to while receiving the sessions at home. One participant mentioned, “I used to keep my health file with me when receiving her call. Everything was mentioned in the health file, so it became easy for me to understand what the therapist was referring to” (IDI-M06).

## Discussion

Our study investigates participants’ and research therapists’ preferences for the delivery methods (face-to-face sessions and phone sessions) of a CBT-based psychosocial intervention for prenatal anxiety. Most participants favored face-to-face sessions due to their ability to build rapport, enhance communication, and facilitate comprehension. However, barriers such as venue accessibility, childcare responsibilities, and lack of family support hindered engagement. Conversely, telephone sessions appealed to some participants as they allowed flexibility in scheduling and the comfort of being at home, but challenges included phone unavailability, poor phone connections, shorter attention spans, and limited mental health awareness of their family members, which posed potential obstacles. Notably, a blend of face-to-face and telephone sessions emerged as the most preferred approach, primarily driven by the rapport established during initial in-person sessions, which carried over into subsequent telephone interactions.

The majority of participants in our study valued face-to-face sessions for their efficacy in establishing rapport with therapists. This adds to the evidence from existing literature, highlighting the advantages of face-to-face therapy sessions and emphasizing their capacity to rapidly build rapport through enhanced verbal and nonverbal communication, ultimately strengthening the therapeutic relationship (Del Giacco et al., 2020). On the contrary, telephone-based relationships may pose challenges to establishing a therapeutic alliance (Haas et al., 1996; Brenes et al., 2011). Without access to nonverbal cues, therapists may find it difficult to grasp clients’ experiences and convey empathy effectively (Miller, 1973; Bennett, 2004; Mozer et al., 2008). Notably, most of our participants expressed greater comfort in disclosing personal information during face-to-face sessions compared to telephone sessions. Although most studies report no significant differences in patient or therapist ratings of information disclosure (Brown, 1985; Spizman, 2001), a patient self-assessment questionnaire revealed that 78% felt their counselor better understood and supported their self-exploration in face-to-face interactions than in telephone conversations (Antonioni, 1973).

Both therapists and participants agreed that face-to-face sessions promoted a deeper understanding of the intervention’s content and associated activities in comparison to the telephone sessions. This enhanced comprehension may be due to the ability to pick up on nonverbal cues during the sessions (Miller, 1973; Bennett, 2004). In the original conception of HMHB, we envisioned it would be delivered face-to-face. The protocol incorporated pictorial illustrations designed to help participants challenge and replace unhelpful thought patterns and behaviors with helpful thoughts and behaviors, promote in-depth discussions for mental health awareness, and active participation in relaxation exercises that would be guided by the therapist during face-to-face sessions. Another notable feature of the intervention was family involvement, which therapists found more feasible during in-person therapy.

Some participants expressed a preference for telephone sessions, especially those living in nuclear family households and in environments that were not prone to disruptions. Phone sessions offered scheduling flexibility, easy rescheduling, and the convenience of avoiding travel to the session venue. A recent study examining attitudes toward internet-delivered CBT found that convenience and time flexibility were frequently cited as primary reasons for this preference (McCall et al., 2020).

The therapists reported that phone sessions were relatively shorter compared to face-to-face sessions, which they attributed to the less serious attitudes of the participants when receiving sessions by phone, as well as their shorter attention spans during phone-based interactions. While existing evidence supports the notion that phone sessions are generally shorter than face-to-face ones (Irvine et al., 2020), findings are contradictory regarding recipients’ attentiveness. Some studies reported no significant difference in attentiveness (Spizman, 2001; Stephenson et al., 2004), while others noted reduced attentiveness due to the absence of visual cues or due to the perceptions of telephone psychotherapy as being less important (Mozer et al., 2008). This perception of not being taken seriously during phone sessions was also reported by therapists in our study.

Interestingly, a recent systematic review of 15 studies investigating the interactional differences between telephone and face-to-face psychological therapy found no significant disparities in therapeutic alliance, disclosure, empathy, attentiveness, or participation (Irvine et al., 2020). However, our participants’ preference for face-to-face or phone sessions was notably influenced by practical and emotional barriers. Those favoring phone sessions encountered difficulties stemming from a lack of family support, including the absence of an escort, typically a close male relative or elder female, to accompany them to hospital appointments in line with prevailing family norms, as well as a lack of financial resources to cover travel costs. Among those who successfully attended face-to-face sessions, some faced challenges in maintaining full concentration due to childcare responsibilities and family pressures. In other qualitative studies conducted with similar populations, the issues of inadequate family support and lack of money to pay for transportation have been consistently highlighted as barriers to receiving interventions (Atif et al., 2015; Nakku et al., 2016; Rowther et al., 2020; Nazir et al., 2022; Atif et al., 2023).

Many participants who favored face-to-face sessions were women who encountered significant obstacles related to the unavailability of a personal mobile phone. This issue is underscored by a UNESCO report highlighting Pakistan’s substantial gender gap in mobile phone ownership, with Pakistani women being 38% less likely than men to own a mobile phone and 49% less likely to use mobile internet (UNESCO, 2023). Given that a majority of our participants came from lower socioeconomic backgrounds, they had to depend on male family members’ mobile phones to receive the sessions, which were often unavailable to them during normal working hours. Furthermore, even when mobile phones were accessible, technical issues, such as poor call quality and frequent call drops, could disrupt the sessions. Both therapists and participants acknowledged this challenge as a major barrier to phone-based sessions.

Additional challenges encountered during phone sessions, as reported by certain participants, included a lack of privacy due to overcrowded households and suspicion from family members regarding extended conversations with the “therapist,” reflecting a broader issue of limited mental health awareness. These individuals valued face-to-face sessions as safe spaces for sharing concerns without fear of disclosure, eavesdropping by family members, or stigmatization. Notably, a review of RCTs using telephone-delivered CBT identified challenges such as limited control over the environment and potential threats to privacy and confidentiality (Brenes et al., 2011).

It is worth highlighting that among participants who preferred telephone sessions, a significant number attributed their positive experiences to the initial face-to-face contact with their therapist. They felt that this initial in-person interaction played a pivotal role in establishing rapport, which, in turn, facilitated the effective delivery of subsequent phone-based sessions. This finding aligns with an online survey conducted among 514 individuals, indicating that people who combine face-to-face and online psychotherapy perceive it as more efficient with fewer access barriers compared to face-to-face therapy (Sora et al., 2022).

One notable strength of our study is that data were collected from both the trial participants and the research therapists, enabling us to enhance the thoroughness of our inquiry. Additionally, the deliberate selection of participants through purposive sampling, of those who did and did not complete all the therapy sessions, broadened the range of insights gathered from these interviews. Moreover, two members of the research team were independently engaged in creating the codebook, enhancing the reliability and validity of the coding framework and increasing the robustness and objectivity.

There were several limitations to our study. The research took place in a tertiary hospital situated in a semi-urban region, with fairly homogeneous demographic characteristics. As a result, the implications drawn from the study may not be applicable to a broader population. Another limitation arises from the fact that certain interviews were conducted via telephone, potentially restricting the establishment of rapport and the exchange of information between the interviewer and the interviewees. Moreover, the study was conducted during the COVID-19 pandemic, which could have influenced participants’ perceptions and experiences and may not fully represent the post-pandemic scenario. The research focused only on antenatal anxious women, and further research is needed to explore the experiences of face-to-face vs. telephonic sessions outside of the perinatal period.

## Conclusion

The findings of this research emphasize the importance of implementing flexible intervention delivery strategies that take into account cultural norms, logistical obstacles, and individual family dynamics. To promote mental well-being among pregnant women in Pakistan, we recommend the adoption of a mixed approach that combines the strengths of both face-to-face and phone-based interventions. This approach allows for a more personalized and adaptable form of care, and can better support pregnant women in managing anxiety during pregnancy and improving their overall mental health.

## Data Availability

The data that support the findings of this study are not publicly available due to participant confidentiality restrictions but are available from the corresponding author upon reasonable request.
