# Supplementary material for: Exploring preference for delivery methods for a psychosocial intervention for prenatal anxiety: A qualitative study from a tertiary care hospital in Pakistan
Source: Glob Ment Health (Camb). 2024 May 9;11:e66. doi: 10.1017/gmh.2024.59 (PMC11140489; doi:10.1017/gmh.2024.59)
Supplement: Peer review history

# Editor report: Recommendation: Exploring preference for delivery methods for a psychosocial intervention for prenatal anxiety: A qualitative study from a tertiary care hospital in Pakistan — R0/PR2

**DOI:** 10.1017/gmh.2024.59.pr2

**Affiliations:** University of KwaZulu-Natal, South Africa

Please attend to the reviewers' concerns before the paper can be accepted.

# Editor report: Decision: Exploring preference for delivery methods for a psychosocial intervention for prenatal anxiety: A qualitative study from a tertiary care hospital in Pakistan — R0/PR3

**DOI:** 10.1017/gmh.2024.59.pr3

**Affiliations:** Johns Hopkins University Bloomberg School of Public Health, United States

No accompanying comment.

# Editor report: Recommendation: Exploring preference for delivery methods for a psychosocial intervention for prenatal anxiety: A qualitative study from a tertiary care hospital in Pakistan — R1/PR5

**DOI:** 10.1017/gmh.2024.59.pr5

**Affiliations:** University of KwaZulu-Natal, South Africa

No accompanying comment.

# Editor report: Decision: Exploring preference for delivery methods for a psychosocial intervention for prenatal anxiety: A qualitative study from a tertiary care hospital in Pakistan — R1/PR6

**DOI:** 10.1017/gmh.2024.59.pr6

**Affiliations:** Johns Hopkins University Bloomberg School of Public Health, United States

No accompanying comment.
